# Linking Ethanol-Addictive Behaviors With Brain Catecholamines: Release Pattern Matters

**DOI:** 10.3389/fnbeh.2021.795030

**Published:** 2021-12-16

**Authors:** Vladimir P. Grinevich, Evgeny M. Krupitsky, Raul R. Gainetdinov, Evgeny A. Budygin

**Affiliations:** ^1^Department of Neurobiology, Sirius University of Science and Technology, Sochi, Russia; ^2^V.M. Bekhterev National Medical Research Center for Psychiatry and Neurology, St. Petersburg, Russia; ^3^Laboratory of Clinical Psychopharmacology of Addictions, St.-Petersburg First Pavlov State Medical University, St. Petersburg, Russia; ^4^Institute of Translational Biomedicine and St. Petersburg State University Hospital, St. Petersburg State University, St. Petersburg, Russia

**Keywords:** addiction, motivation, dopamine, norepinephrine, phasic and tonic release, optogenetics

## Abstract

Using a variety of animal models that simulate key features of the alcohol use disorder (AUD), remarkable progress has been made in identifying neurochemical targets that may contribute to the development of alcohol addiction. In this search, the dopamine (DA) and norepinephrine (NE) systems have been long thought to play a leading role in comparison with other brain systems. However, just recent development and application of optogenetic approaches into the alcohol research field provided opportunity to identify neuronal circuits and specific patterns of neurotransmission that govern the key components of ethanol-addictive behaviors. This critical review summarizes earlier findings, which initially disclosed catecholamine substrates of ethanol actions in the brain and shows how the latest methodologies help us to reveal the significance of DA and NE release changes. Specifically, we focused on recent optogenetic investigations aimed to reveal cause-effect relationships between ethanol-drinking (seeking and taking) behaviors and catecholamine dynamics in distinct brain pathways. These studies gain the knowledge that is needed for the better understanding addiction mechanisms and, therefore, for development of more effective AUD treatments. Based on the reviewed findings, new messages for researches were indicated, which may have broad applications beyond the field of alcohol addiction.

## Introduction

Alcohol use disorder (AUD) is a problematic pattern of alcohol use, which is widely spread among adult population in the world and for the most part accompanied by co-occurring psychiatric disorders, e.g., drug use disorders, major depressive disorders, specific phobias, and antisocial and borderline personality disorders (Kranzler and Soyka, 2018), and results in serious health and socio-economic problems in our society (Substance Abuse and Mental Health Services Administration (Samhsa), 2019; Tucker et al., 2020). AUD exerts multiple features which include compulsive alcohol seeking and aberrant control over drinking resulting in excessive ethanol consumption, craving, withdrawal syndrome, commonly occurring relapse and is stated a chronic disease (Nieto et al., 2021). Clarification of neurobiological substrates of ethanol and their modification induced by chronic ethanol ingestion is critical in understanding the clinical manifestation of AUD and to the development of effective treatments (Kranzler and Soyka, 2018). Unfortunately, our knowledge of the precise neurochemical mechanisms responsible for the development and progression of AUD is still incomplete. The functioning of dopamine (DA) and norepinephrine (NE) systems seems to be altered by ethanol more than others and, therefore, they were the main targets in the field of alcohol research.

The effect of ethanol to increase the concentration of DA in the mesolimbic system has been known since the late 1980s (Imperato and Di Chiara, 1986). To date, the studies using animal models have provided advanced characterization of the DA implication in neurobiological effects of ethanol, while clinical studies have concurred with a clarification of the involvement of DA in AUD (Charlet et al., 2013; Söderpalm and Ericson, 2013; Chen et al., 2018; Salinas et al., 2021). On the other hand, both animal and human studies have also demonstrated that dysregulation of the NE system, which is tightly linked with arousal and stress response, can also be a key component of the AUD pathophysiology (Haass-Koffler et al., 2018). NE neuron cell bodies are clustered in discrete nuclei in the brainstem and pons, whose axons project widely throughout the brain including numerous regions involved in stress, reward and ethanol-related behaviors. The largest and best characterized of these nuclei is the locus coeruleus (LC), which projects to the amygdala and cortex to promote cognitive and sensory processing, and to pave the way for anxiety and stress responses (Sara, 2009; Szabadi, 2013). Noradrenergic afferents from the LC to the ventral tegmental area (VTA) have been shown to modulate DA neuron firing and extracellular DA concentrations in the nucleus accumbens (NAc), caudate, and prefrontal cortex (Mejias-Aponte, 2016), likely via activation of α1-adrenoreceptors (Rommelfanger et al., 2009). In fact, it has been observed in both preclinical and clinical studies that selective antagonists of α1-adrenoreceptors and agonists α2-adrenoreceptors alter ethanol- seeking and consumption (Haass-Koffler et al., 2018). In general, drugs designed to reduce central NE activity appear to decrease motivation to ethanol and drinking behaviors in rodents, particularly ethanol-preferring animals (Haass-Koffler et al., 2018).

Furthermore, many mood and stress-associated disorders are comorbid with alcohol- addictive behaviors (Grant, 2004; West et al., 2016; Gilpin and Weiner, 2017). In fact, individuals identified with post-traumatic stress disorder are more likely to develop an AUD (Kessler et al., 1997). It appears that ethanol is a common tool to deal with stress and anxiety-associated conditions due to its anxiolytic effect. However, withdrawal from chronic drinking can increase anxiety and other stress-related symptoms. Consequently, although stress events promote excessive ethanol intake, excessive drinking may deteriorate symptoms of stress disorders (Gilpin and Weiner, 2017). It is why, the successful development of more effective treatments of such pathological conditions will require not only a better understanding of the specific mechanisms responsible for ethanol’s positive reinforcing actions but also the mechanisms linking negative reinforcement and AUD. In fact, the activation of brain stress systems is hypothesized to be a key element of the negative emotional state of alcohol dependence (or any other addictive substance) when ethanol seeking and taking are driven by negative reinforcement mechanisms.

Over the past several decades the development and use of translational animal models has resulted in notable progress in our understanding of ethanol and stress induced effects on synaptic transmission, especially on DA and NE systems. Perhaps, more importantly, these findings provided critical insights into some of the neural circuits that are involved in ethanol addiction-related behaviors, which include the VTA—NAc and LC—prefrontal cortex and basolateral amygdala pathways (Vena et al., 2020; Ye et al., 2020). However, it is required to not only describe the changes that are apparent following ethanol exposure with or without stress, but also to determine if these alterations play a causal role in the triggering of addictive behaviors. These experiments necessitate the ability to manipulate activity of discrete populations of neurons with precise temporal (ms) and spatial (μm) resolutions during addictive behaviors. Therefore, the use of techniques, which allow us to perform such manipulations as well as to confirm expected neurotransmitter dynamics *in vivo* is essential.

Recent studies clearly demonstrated that the combination of ethanol self-administration paradigms with optogenetics and real-time electrochemical recordings are extremely helpful in this regard. Though alcohol researchers are only in the beginning stages of using these approaches, latest findings have already provided new insights into the determinant role of specific neural circuits in ethanol-seeking and ethanol-drinking behaviors.

This review aimed to highlight how latest methodological developments help us to understand the significance of differences in catecholamine release patterns within distinct pathways for the triggering and suppressing of ethanol-addictive behaviors.

## Acute Ethanol Exposure and Brain Catecholamine Changes

Researchers have long pursued investigation of the mechanisms of ethanol action in the brain using preferentially rodent models. It was found that ethanol influences CNS functioning by modifying an activity of many neurotransmitter systems, including γ-aminobutyric acid (Koob, 2004; Siggins et al., 2005), glutamate (Woodward, 2000), serotonin (Grant, 1995), NE (Weinshenker et al., 2000), neuropeptide Y (Thiele et al., 1998), vasopressin (Edwards et al., 2012), adenosine (Nam et al., 2013) and DA (Weiss and Porrino, 2002; Gonzales et al., 2004; Koob, 2013). However, the mesolimbic DA appears to be modified in its functioning more than others in all addictions and appears to fluctuate differently and predictably, regardless of abused substance (Melis et al., 2005). This circuit is comprised of the dopaminergic neurons in the VTA and their projections to the NAc (and several other brain regions) and closely associated with activation of the reward system. Therefore, DA transmission received prominent attention in the alcohol research field.

The studies on rodents clearly demonstrated that acutely administered ethanol enhances the firing rate of VTA DA neurons (Brodie, 2002), increasing DA release from dopaminergic terminals (Imperato and Di Chiara, 1986; Tang et al., 2003) similarly to other abused drugs. At the same time, DA uptake parameters measured by voltammetry *in vivo* and *in vitro* remain unaffected (Budygin et al., 2001a,b, 2005; Jones et al., 2006). It has been appreciated that ethanol actions on DA cells in the VTA might be mediated by its first metabolite acetaldehyde (AcH), which directly increases the activity of these cells (Foddai et al., 2004). Moreover, AcH in the brain can enzymatically interact with DA to synthesize tetrahydro-isoquinoline alkaloid salsolinol (SAL), which level was shown to increase significantly in terminals of dopaminergic cells in the striatum and hypothalamus following ethanol exposure (Matsubara et al., 1987; Naoi et al., 2002; Mravec, 2006; Chen et al., 2018). It has also been indicated that ethanol administration enhances the reaction of DA with dopaldehyde (the monoamino oxidase product of DA) to form tetrahydropapaveroline (THP), a biological precursor of morphine (Sango et al., 2000; McCoy et al., 2003). SAL and THP have been reported to mediate certain effects of ethanol (Myers, 1996; McCoy et al., 2003; Mravec, 2006; Deehan et al., 2013; Bassareo et al., 2021). In relation to the reinforcing properties of ethanol, the most intriguing findings have been that SAL and THP infused into the cerebral ventricles of rats or monkeys trigger abnormal consumption of ethanol (Melchior and Myers, 1977; Myers and Oblinger, 1977; West et al., 1998) and that SAL shows off high affinity to D_2_/D_3_ receptors (Patsenko et al., 1987; Rodd et al., 2003). Furthermore, microinjections of THP into the NAc cause an intense preference for ethanol in rats (Duncan and Fernando, 1991). It was also shown that SAL infused locally into different areas of the rat brain profoundly increased the extracellular DA in these regions. Remarkably, the potency of SAL to increase the output of DA in the striatum was significantly stronger compared to that of methamphetamine (Nakahara et al., 1994).

According to PET studies, ethanol triggers DA release in the NAc of the human brain (Boileau et al., 2003). It is important to highlight that this neurotransmitter can be released in the terminal field in two distinctive firing patterns: tonic, that is a single spike activity at a frequency of ∼5 Hz, and phasic, that appears as synchronized bursts of action potentials at higher frequencies (≥20 Hz) (Grace and Bunney, 1983b; Grace, 1991; Schultz, 1998; Wightman and Robinson, 2002; Anstrom et al., 2009). These patterns produce discrete modes of DA release, resulting in DA concentration changes lasting from seconds to minutes (Wightman and Robinson, 2002). Specifically, tonic firing results in low steady-state DA extracellular concentrations that are usually lower than 50 nM (Parsons and Justice, 1992), while phasic firing produces relatively large, transient increases in DA levels, which may significantly exceed 50 nM in the extra neuronal space (Grace and Bunney, 1983a,b,c, 1984a,b; Freeman et al., 1985; Wightman and Zimmerman, 1990; Hyland et al., 2002; Wightman and Robinson, 2002). Importantly, these two modes of DA transmission appear to operate different functions that may result in dissimilar behavioral consequences, though some overlap is also possible. Ethanol alters both tonic and phasic DA fluctuations in the NAc measured by microdialysis and fast-scan voltammetry, respectively (Robinson et al., 2009). However, the role of tonic and phasic DA release in ethanol-seeking and -drinking behaviors remained unknown until recently.

Significantly less attention has been focused on the involvement of noradrenergic transmission at acute ethanol action in the brain. Borg et al. (1981) demonstrated that the NE turnover was increased in alcoholics and healthy controls. Increased turnover of the brain NE following an acute dose of ethanol was also reported in rats (Corrodi et al., 1966; Hunt and Majchrowicz, 1974; Smith et al., 1985).

More accurate assessment of acute effects of ethanol on central NE transmission was made by employing microdialysis technique in freely moving rats. It was found that at a low dose (0.2 mg/kg, i.p.) ethanol increased, while at higher dose (2.0 mg/kg, i.p.) decreased extracellular NE concentration in rat frontal cortex (Murphy et al., 1983; Rossetti et al., 1992). It was speculated that the decrease in NE efflux may be associated with the sedative-hypnotic properties of ethanol at high doses, whereas the elevated NE may represent a neurochemical correlate of the arousal and increased alertness elicited by low doses of ethanol. It was also shown that NE levels were elevated in the NAc (Karkhanis et al., 2014) and basolateral amygdala (Karkhanis et al., 2015) of socially isolated but not in naïve rats following the 2 g/kg dose. It was suggested that these changes might promote the escalated ethanol-drinking behavior observed in rats subjected to chronic adolescent stress (Karkhanis et al., 2015). The study focused on NE transmission in the bed nucleus of stria terminalis indicated that ethanol (0.5, 1.0 g/kg, i.p.) dose-dependently increased NE levels in dialysates (Jadzic et al., 2021).

In contrast to the effect on DA cell bodies, ethanol administration (1–2 g/kg) directly onto a single neuron in the rat LC produced a suppression in the firing rate (Strahlendorf and Strahlendorf, 1983). In line with this observation, recordings in brain slices revealed the decreased spontaneous firing rate of LC cells, suggesting that acute ethanol exposure might suppress NE release (Verbanck et al., 1990). This effect could explain the decreases in NE concentrations measured by microdialysis in terminal fields following high doses of ethanol (see above). Collectively, these results indicate that acute ethanol has a powerful action on catecholamine dynamics that suggests profound adaptations in both DA and NE brain systems following its chronic exposure.

## Chronic Ethanol Exposure and Brain Catecholamine Changes

The acute effects of abused substances often specify knowledge of their initial target in the brain but don’t give relevant information on alteration related to the phenomenon of the development of addictive behaviors. In fact, the outcomes of chronic ethanol exposure on catecholamine dynamics are more multifaceted. Overall, in contrast to acute action, chronic ethanol exposure and withdrawal result in hypodopaminergic state (Weiss and Porrino, 2002; Koob, 2003; Melis et al., 2005). This condition is considered as one of the important causes that triggers a seeking and taking compounds of abuse including ethanol after the prolonged exposure to them (Koob, 2003; Melis et al., 2005; Diana, 2011). The low levels of DA were found in the caudate nucleus of alcoholic brains (Noble, 1996). In animal models, a profound reduction of spontaneous firing rate as well as a burst firing of the identified NAc-projecting VTA DA neurons in rats (Diana et al., 1993b) and mice were revealed (Bailey et al., 2001). These alterations induced by chronic ethanol exposure can explain substantial decreases in extracellular DA levels in the rat NAc measured by microdialysis (Rossetti et al., 1992; Diana et al., 1993a; Barak et al., 2011). Importantly, original accumbal DA concentrations were found to be recovered when ethanol was self (Weiss et al., 1996) and passively administered (Diana et al., 1993a,^1996^). Furthermore, intracranial self-stimulation (ICSS) studies demonstrated that ethanol-withdrawn rats can maintain the ICSS behavior with the increased current intensity (Schulteis et al., 1995). Therefore, it could be speculated that the neural substrate responsible for maintaining the ICSS behavior is hyperpolarized or more refractory in the ethanol-dependent subject as compared with naïve control (Diana, 2011). However, an electrical stimulation is not specific for DA cells only, therefore, the involvement of other neurotransmitter systems cannot be completely ruled out under this circumstance.

Furthermore, the acceleration in striatal DA uptake was detected by voltammetry *in vitro* in ethanol drinking monkeys and rats (Budygin et al., 2003; Deal et al., 2018), and in rats (Budygin et al., 2007) and mice (Karkhanis et al., 2015) chronically exposed to ethanol vapor. In agreement with voltammetry studies, an enhanced DA transporter (DAT) protein levels have been found following chronic ethanol treatment (Rothblat et al., 2001). Therefore, an altered reuptake of the neurotransmitter may also contribute to the decrease in extracellular DA concentrations observed under the condition of chronic ethanol exposure.

There are some lines of evidence that chronic drinking may increase the sensitivity of the posterior VTA to the reinforcing effects of ethanol (Rodd et al., 2005a,b). The increased excitation of DA neurons following chronic ethanol exposure (Brodie, 2002) can explain the greater accumbal DA release in response to acutely administered ethanol (Ding et al., 2009). The latest study revealed that phasic but not tonic stimulation induced a higher DA efflux in the NAc core of high drinking mice when compared to ethanol naïve controls (Liu et al., 2020).

Presynaptic dopamine D2 receptors (D2R), which are primarily involved in the control of DA release, can be altered following chronic ethanol exposure as well (Hietala et al., 1994; Volkow et al., 1996, 2006, 2010; Tupala et al., 2001). Using positron emission tomography in the brain of individuals with addictions to diverse substances (ethanol, opioids, cocaine, and amphetamine) revealed that they have a reduced D2R availability in reward regions (Volkow et al., 2010). Conversely, transfection of RNA encoding the D2R into the brain of rats, significantly increasing its expression, is associated with a significant decrease in ethanol consumption in alcohol-dependent rats (Thanos et al., 2001). These findings support the hypothesis of the significant role DA neurotransmission might play in the mechanism underlying alcohol dependence, and may be the first steps toward an eventual genetic treatment for AUD.

Scrupulous consideration should be given to DA dynamics, which are coincidental with the initiation and with carrying out of alcohol self-administration. Several studies have explored how DA changes correlate with appetitive and consummatory phases of ethanol intake. A well-designed microdialysis experiment has found a significant rise in DA concentration in the NAc of rats during their placement into the operant chamber and in the first several minutes of ethanol consumption (Doyon et al., 2003). However, the lever pressing that reflects a motivation for ethanol solution did not alter DA levels. It should be clarified that a microdialysis is the best suitable approach to evaluate changes in tonic DA release, because this technique has a relatively slow temporal resolution. Therefore, undeviating DA measures obtained with microdialysis disguise alterations in DA release, which can take place on subsecond time scale. Indeed, the latest fiber photometry studies revealed fast DA increases in rat NAc across different stages of ethanol self-administration. Remarkably, these DA transients were subregion-specific (Liu et al., 2020).

A long-term ethanol consumption finally leads to terminal stages of AUD when negative emotional stage is predominant, especially during alcohol withdrawal. This condition appears at least in part due to hypodopaminergic, whereas hyperactivation of NE system triggered by different stress factors may play a supplementary role. Indeed, increased central NE signaling and increased sympathetic activity have been well documented in alcoholics, particularly during onset of withdrawal when elevated NE levels in cerebrospinal fluid (Hawley et al., 1981) and in plasma (Patkar et al., 2003) were observed (Fitzgerald, 2013; Haass-Koffler et al., 2018).

There is also emerging evidence that targeting the NE system may be beneficial in the treatment of AUD, with prazosin showing the most promise. The α1 receptor antagonist, prazosin, appeared efficacious in decreasing stress- and cue-induced alcohol craving and in normalizing the stress dysregulation associated with early withdrawal in alcohol dependent individuals (Simpson et al., 2009; Fox et al., 2012; Kenna et al., 2016). Another α1-blocker doxazosin was also introduced for AUD due to demonstrating more favorable pharmacokinetic profile than prazosin, and being selectively effective in Alzheimer’s disease patients with high family history density of alcoholism. There were also evidences that α2-agonists such as lofexidine, clonidine or a newer α2-agonist dexmedetomidine, may have an adjunctive role in reducing sympathetic overdrive for alcohol withdrawal management (Walinder et al., 1981; Cushman et al., 1985; Darrouj et al., 2008).

As of investigation of rats chronically exposed to high-dose ethanol, administration of α_1_-adrenergic receptor blockade prazosin (Walker et al., 2008) or the β-adrenergic receptor blocker propranolol (Gilpin and Koob, 2010) abolished accumulation of ethanol intake during withdrawal.

During withdrawal from chronic binge-like ethanol exposure, elevated sympathetic activity contributes to aversive somatic symptoms, thus promoting relapse in ethanol drinking behavior (Heilig et al., 2010). In rat studies, blockade of α1- or β-adrenergic receptors (Trzaskowska et al., 1986; Riihioja et al., 1997) or activation of α2-adrenergic autoreceptors (Riihioja et al., 1997) reduced alcohol withdrawal symptoms such as convulsions, tremors, and locomotor hyperactivity. It has also been shown in rats, that blockade of NE signaling during alcohol withdrawal attenuated ethanol drinking (Walker et al., 2008).

Therefore, DA and NE transmission can be profoundly influenced by chronic ethanol exposure. These changes indicate a disbalance between DA and NE systems that is characterized by the DA hypofunction and hyperfunctoning of the NE system. Moreover, some correlative data suggest an importance of released catecholamines in certain brain regions under the condition of prolonged ethanol drinking. However, the precise role of DA and NE release changes in addictive behaviors was unclear until the use of optogenetic tools combined with satisfied ethanol self-administrative paradigms. The next section summarized the latest findings, which revealed how ethanol-seeking and -drinking behaviors are causally shaped by these catecholamines.

## From Brain Concentration Levels to Release Patterns

Revealing alterations in neurotransmitter concentrations, especially coupled with the initiation and maintenance of ethanol consumption, is an important first step toward understanding the role of certain brain systems in the development of addictive behaviors. However, the challenge is to determine whether observed changes perform a causal job in ethanol seeking (appetitive behavior) and intake. Fortunately, recent technological advances such as optogenetics now provide the opportunity to answer these critical questions (Tsai et al., 2009; Adamantidis et al., 2011; Witten et al., 2011; Bass et al., 2013). Indeed, this approach allowed us to precisely mimic certain patterns of neurotransmission in distinct circuitries in behaving animals (Bass et al., 2013; Mikhailova et al., 2016; Budygin et al., 2020; Deal et al., 2020). Therefore, a far more complete exploration of the role of brain neurotransmitters in ethanol-addictive behaviors has become possible.

Ethanol-drinking behavior can be broken down into two discrete components. Appetitive (motivational) component is involved into reward seeking behaviors directly, while consummatory component is involved into behaviors associated with the concrete action of drinking. These components can be differentially regulated. and dysregulation of both these behaviors may contribute to the development and further escalation of AUD. To explore the role tonic and phasic patterns of DA release play in consummatory behavior, optogenetics has been initially used to selectively shape DA dynamics in the NAc of rats during voluntary ethanol drinking in intermittent two-bottle choice procedure (Bass et al., 2013). In this well established procedure, rats had access to 20% ethanol and water 3 times per week (Wise, 1975; Simms et al., 2008; Moorman and Aston-Jones, 2009). Subjects, which demonstrated high stable levels of ethanol intake during first 7 weeks, were injected into the VTA with the viral construct with the tyrosine hydroxylase promoter that restricted expression of channel rhodopsin 2 (ChR2) to DA cells. Therefore, the applied design allowed to explore the effects of optogenetic activation of the DA neurons in the VTA on the voluntary intake of ethanol in pharmacologically meaningful doses. The experiments have indicated that ethanol consumption can be differently affected by tonic and phasic patterns of VTA DA cell activation without a significant effect on water intake. Thus, a substantial delay to the first lick and a reduction in the total amount of consumed ethanol were found when VTA DA cells were optogenetically activated using a low frequency (5 Hz), which triggers tonic release pattern (Bass et al., 2013). Importantly, the same protocol of stimulation had no effect when applied in home cage for a 10 min before the drinking session. It has been suggested that increased tonic DA activity was through a dopamine D2 autoreceptor-mediated feedback mechanism in order to suppress phasic DA release (Grace, 2000; Phillips et al., 2003; Budygin et al., 2020). It has been known that phasic DA release is necessary for the capability of reward predictive cues to trigger behaviors directed toward obtaining the reward (Steinberg et al., 2013). Remarkably, the drinking paradigm employed in that study promoted binge-like ethanol drinking during the first 5–10 min of each session was coincident with the placement of rats in the chambers along with the presentation of ethanol-associated cues. Therefore, optogenetically pushing DA into a tonic pattern of transmission may preclude phasic signaling naturally triggered by the contextual cues coupled with the drinking environment. Outstandingly, ethanol solution remained available for the last 20 min of each session, therefore rats had the opportunity for a compensation of unconsumed ethanol solution during the stimulation. Nevertheless, rats did not compensate for their loss when optical stimulation was terminated. One possible explanation for this finding could be that optogenetically-induced tonic DA increase can imitate the pharmacological effect of ethanol on DA neurotransmission. Under this circumstance, subjects would need a lower dose to obtain the expected intoxicating effects. However, the same stimulation applied in the home cage had no effect on ethanol intake. Consequently, the suppression of cue-induced phasic DA release associated with the onset of a drinking session may affect ethanol drinking even after the optostimulation was terminated.

No significant changes in drinking behaviors, including the latency to the first lick and total amount of consumed ethanol, were observed in the study following phasic stimulation. It was possible that rats were already habituated to the procedure and consumed quite high levels of ethanol during drinking sessions. Thus, the incapability of the stimulation to escalate ethanol-drinking behaviors might be due to ceiling effect. Likely, because subjects were already greatly motivated to obtain and consume ethanol, further optogenetic enhancement of phasic DA signaling was not effective. It should be noticed that ethanol seeking and consummatory measures are not interconnected (Samson and Czachowski, 2003; Siggins et al., 2005; Budygin et al., 2020). In fact, a meta-analysis of the drinking behavior of 234 rats revealed no correlation between prior day ethanol intake and extinction probe trial responding (Samson and Czachowski, 2003). Furthermore, infusion of raclopride, a D2R antagonist, into rat NAc selectively inhibited lever-press responding for ethanol without effect on its consumption (Samson and Chappell, 2004). Consequently, shifting DA transmission in the NAc into a phasic mode may primarily promote ethanol-seeking (motivational) behavior with no changes in the intake. In fact, employing an operant drinking behavioral test is required to make conclusions regarding motivational changes.

It should be mentioned that another optogenetic study reported decreased ethanol-drinking behaviors in high drinking mice following the enhancing of the VTA DA neuron burst activity, while the tonic protocol stimulation was ineffective (Juarez et al., 2017). However, the stimulation parameters were distinct from those, which were used in the first study, while the generated DA release patterns were not validated by the measurements of DA release. Furthermore, optoactivations of DA cells in the VTA and NAc were not performed during drinking sessions. This fact may raise a question about a direct cause-effect relationship between neurochemical and behavioral consequences. On the other hand, the results obtained in the study on mice may reveal DA plasticity changes, opening a new avenue for exploration of long term effects of optogenetic interventions.

**FIGURE 1:**
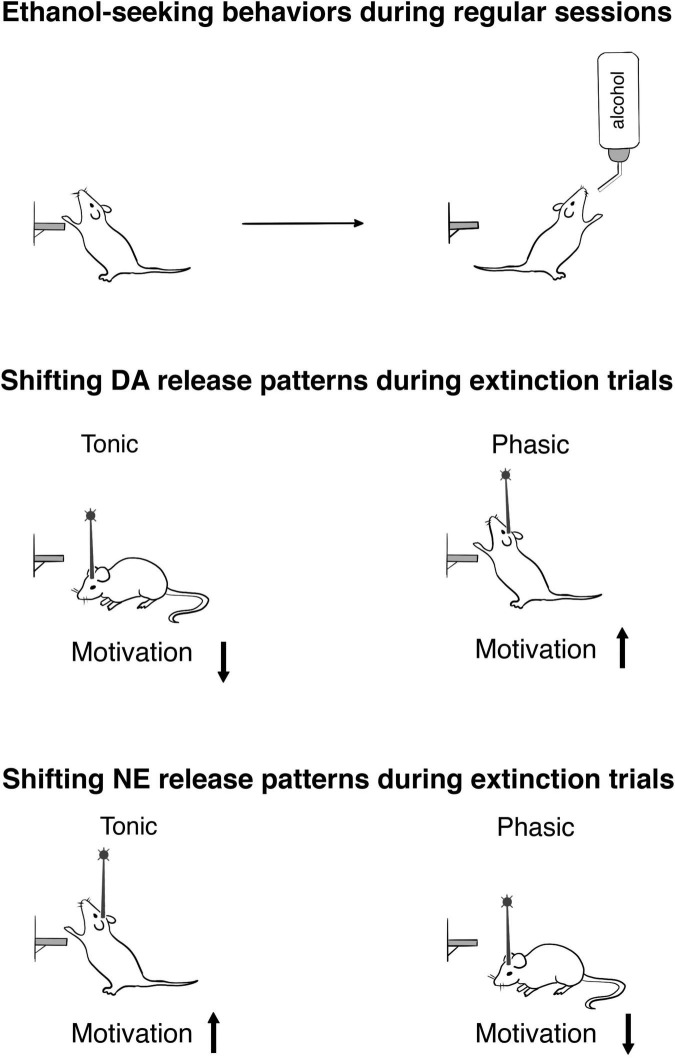
Optogenetic exploration of ethanol-seeking behaviors in rats. The upper panel schematically demonstrates the completion of the lever press response requirement (30) during reinforced session. To obtain an appetitive measure devoid of any consummatory behaviors, extinction trials were conducted. The middle panel shows how optogenetic shifting of the VTA-nucleus accumbens DA release into the tonic and phasic patterns affects the motivation to obtain ethanol during extinction trials (for details, see Budygin et al., 2020). The lower panel indicates motivational changes resulting from the patterns of the LC-NE release (for details, see Deal et al., 2020).

Notably, these first studies that sought to establish a causal role of mesolimbic DA signaling in ethanol drinking (Supplementary Table 1) employed a method that did not permit for the discrete evaluation of the seeking (or motivational) component of the ethanol-taking behavior (Bass et al., 2013; Juarez et al., 2017). Therefore, the next step was to address this gap by integrating an optogenetic approach and an operant ethanol self-administration regimen that could separate motivational (appetitive) and consummatory measures in order to reveal how accumbal DA release dynamics influence the motivation to obtain ethanol (Figure 1). It should be pointed out that the chosen parameters of optostimulations to mimic tonic and phasic increases in DA transmission in behaving animals were used previously by our (Bass et al., 2013; Mikhailova et al., 2016) and other groups (Tsai et al., 2009; Adamantidis et al., 2011). The release patterns were confirmed by the use of real-time electrochemical measurements of DA release. Importantly, these stimulation protocols did not result in any behavioral or neurochemical indications of non-physiological conditions. To completely isolate a measure of ethanol seeking from any consummatory behaviors, extinction probe trials were targeted with optogenetic stimulation (Budygin et al., 2020). Subjects were placed into the operant box with all the ethanol-related cues, where the lever could be pressed without limit, but ethanol solution was not provided. These experiments revealed that phasic and tonic VTA-NAc DA release patterns bidirectionally modulate ethanol-seeking behavior, measured as the number of lever presses. Specifically, we found that a high-frequency stimulation pattern that evoked DA transients with temporal and concentration features similar to real-time DA fluctuations, which were observed during drug-seeking behaviors (Phillips et al., 2003; Owesson-White et al., 2009), resulted in an escalation of ethanol seeking (Budygin et al., 2020). In sharp contrast, applying low-frequency stimulation, and therefore shifting DA release into the tonic mode, where cells simultaneously fire at their basal frequency, suppressed this behavior (Budygin et al., 2020).

What is a neurochemical basis for opposing behavioral responses induced by tonic and phasic increases in DA release? Again, one possible hypothesis is that shifting DA in the NAc into a tonic mode could through activation of D2Rs prevent phasic DA efflux that triggers motivational action. For many years, this assumption was based predominantly on pharmacological results (Grace, 2000; Phillips et al., 2003; Oleson et al., 2009). Remarkably, a series of pharmacological studies have found that monoamine stabilizer (-)-OSU6162, a D2R antagonist, which increased tonic DA release in the NAc (Feltmann et al., 2016), significantly reduced ethanol-seeking behaviors in rats and suppressed craving measures in alcoholics (Steensland et al., 2012; Khemiri et al., 2015). Recently, the interaction between tonic and phasic patterns of DA release was explored in rat NAc integrating optogenetic activation of the VTA DA cells with fast-scan cyclic voltammetry recordings *in vivo* (Budygin et al., 2020). Both tonic and phasic VTA activation enhanced DA transmission, with the amplitude of DA release being considerably larger with phasic pattern. Nevertheless, simultaneous stimulation with both tonic and phasic modes resulted in a significant reduction of the DA response measured with phasic activation alone (Budygin et al., 2020). Consequently, continuous rise of tonic DA decreased a lever press responding for ethanol by precluding DA axons from triggering phasic signal that should promote the seeking behavior directed to obtain the abused substance. These studies pointed to a novel pharmacological strategy that involved targeting dopamine D2 autoreceptor function involved in the regulation of tonic DA release. Among such molecular targets certain interest represent trace amine-associated receptor 1 (TAAR1) that is known to alter DA neuronal firing rate and accumbal DA release via modulation of dopamine D2 autoreceptor function (Bradaia et al., 2009; Leo et al., 2014; Berry et al., 2017). In fact, it has been observed that mice lacking TAAR1 displayed significantly greater preference for and consumption of ethanol (Lynch et al., 2013). At the same time, TAAR1 agonist RO5263397 suppressed the expression and development of ethanol-elicited behavioral sensitization in normal but not mice lacking TAAR1 (Wu et al., 2020).

Another study using optogenetic inhibition of VTA DA neurons demonstrated a causal role of DA transmission in relapse (Liu et al., 2020). The key findings of this exploration were that there were distinct DA signatures for different forms of relapse. Specifically, relapse provoked by circumstantial signs was distinctly represented by DA system, while relapse evoked by contingent reacquisition was similar to the activity signature of late ethanol self-administration.

Although the recent findings have brought a wealth of knowledge regarding the DA pathway that underlies ethanol positive reinforcing effects, the neural substrates causally connecting negative reinforcement and AUD remained poorly understood. This gap in our knowledge persists despite a growing body of human and animal research highlighting that stress plays a crucial role in the development, progression and relapse to AUD. One critical conclusion from the studies on the role of DA in ethanol-addictive behaviors was that research should focus not only on changes in neurotransmitter concentration or on the strength of neuronal signal but also on the patterns of neurotransmitter release. It should be mentioned that NE transmission has features, which are similar to DA neurotransmission. Indeed, the LC neurons fired with two different patterns: tonic that was irregular but continuous baseline activity and phasic, when cells fired short bursts at higher frequencies (Aston-Jones and Bloom, 1981; Clayton et al., 2004; Aston-Jones and Cohen, 2005).

Following verification that ChR2 expression levels in the LC of drinking rats were high enough to optogenetically evoke NE release in terminal fields, the LC-NE activity was manipulate to reveal how phasic and tonic increases shape ethanol drinking (Deal et al., 2020). Our results demonstrated opposing consequences of these two modes of NE transmission on behaviors. In fact, tonic stimulation of the LC resulted in increased ethanol consumption, whereas phasic stimulation reduced it in regular drinking sessions. Remarkably, electrophysiological studies revealed pattern-specific activity of the LC under stress conditions (Valentino and Van Bockstaele, 2008; Curtis et al., 2012; McCall et al., 2015; Borodovitsyna et al., 2018). Moreover, tonic, but not phasic activation of LC-NE projections increased anxiety-like behaviors (McCall et al., 2017). Therefore, the increase in tonic NE release, that was capable of inducing a stress-associated condition, could promote ethanol-drinking behavior. That finding was in sharp contrast with our results on consequences of tonic stimulation of the VTA-DA on ethanol drinking (Bass et al., 2013). Taken together, these data indicated that two pathways (LC-NE and VTA-DA) played opposite roles in ethanol consumption, although their neurotransmitters released with identical patterns.

The interpretation of the decreased drinking following phasic LC activation is more complicated in comparison with the consequence of tonic increase in NE transmission. Phasic LC stimulation did not induce anxiety (McCall et al., 2015), therefore, an increase in drinking behavior should not be expected. However, phasic activity of the LC-NE was linked with the encoding of salient stimuli and consequent behaviors (Vankov et al., 1995; Sara and Bouret, 2012). Thus, phasic stimulation of rat LC elicited event-associated potentials in the prefrontal cortex (PFC) and could enhance PFC coding of salient stimuli (Vazey et al., 2018). Therefore, we can speculate that optogenetically-increased cortical activity could interfere with attentional processes, which were required for ethanol-seeking behaviors in the drinking chamber environment and subsequently decreased the consumption.

To find how tonic and phasic NE releases influence seeking (motivated) behavior, non-reinforced extinction probe trials were performed (Figure 1). Ethanol-seeking behavior was not changed by tonic stimulation in this experiment. Notably, rats which were exposed to adolescent social isolation that resulted in anxiety and escalated drinking, also did not show significant alteration in this measure (McCool and Chappell, 2009). This finding demonstrated that replicating a stress condition via optogenetic intervention, which induced tonic NE increase, affected ethanol consumption without alteration of motivation. However, seeking behavior was disturbed when phasic patterns of NE release were induced. This suggests that seeking and drinking elements of ethanol-addictive behaviors could be uniformly sensitive to the distractive action of high frequency LC activation. Remarkably, phasic activation of the VTA-NAc pathway escalated ethanol-seeking behavior (Budygin et al., 2020). Consequently, an increase in phasic release within the LC-NE and VTA-DA circuitries caused opposite consequences in regard motivational behavior, as found with ethanol consumption under the influence of stimulation that induced tonic rise (Budygin et al., 2020; Deal et al., 2020).

## Conclusion

Therefore, optogenetic investigations can provide strong leads into the causal mechanisms and neuronal circuitries of recreational and abusive alcohol-drinking behaviors. One important awareness from these studies is that modern exploration should focus on the pattern of neuronal activity, instead of traditional “increase” or “decrease” in neurotransmitter signaling. As discussed above, the increase in neurotransmission can result in opposite behavioral consequences, such as an escalation in ethanol drinking or its suppression, if the release pattern is different. Importantly, to leverage this advantage, optogenetics should be combined with other techniques, such as real-time fast-scan cyclic voltammetry, photometry, and multiunit recordings to reliably monitor neuronal firing rate and neurotransmitter release patterns following optogenetic interventions. Finally, behavioral procedures, which are integrated with optogenetic explorations of ethanol addiction, should distinguish between appetitive (motivational or seeking) and consummatory (taking) components, since these behaviors are shaped by distinct but overlapping neurotransmitter dynamics. Undoubtedly, incorporating optogenetic tools into alcohol research field should reveal neurochemical mechanisms, which are responsible for the development and escalation of pathological alcohol drinking. This knowledge can be critical for the invention of more effective pharmacological and non-pharmacological (for example, transcranial magnetic stimulation) treatments for AUD.

## Author Contributions

VG and EB performed the literature research. VG, RG, and EB designed the manuscript and wrote the first draft. All authors contributed to the article and approved the submitted version.

## Conflict of Interest

The authors declare that the research was conducted in the absence of any commercial or financial relationships that could be construed as a potential conflict of interest.

## Publisher’s Note

All claims expressed in this article are solely those of the authors and do not necessarily represent those of their affiliated organizations, or those of the publisher, the editors and the reviewers. Any product that may be evaluated in this article, or claim that may be made by its manufacturer, is not guaranteed or endorsed by the publisher.

## Supplementary Material

Supplementary Table 1 The findings from studies exploring consequences of optogenetic interventions on ethanol consumption in two different behavioral paradigms. Blue light stimulations with distinct patterns (tonic and phasic) were conducted through chronic implantable optic fibers, which were connected to a laser of 473 nm with a 100-mW power output. The star (*) indicates that intake measures were performed 3 h following optical stimulations conducted in in high ethanol drinking mice in their home cages. In all other experiments, the stimulations were applied within drinking sessions.
