# Supplementary material for: Linking Ethanol-Addictive Behaviors With Brain Catecholamines: Release Pattern Matters
Source: Front Behav Neurosci. 2021 Dec 16;15:795030. doi: 10.3389/fnbeh.2021.795030 (PMC8716449; doi:10.3389/fnbeh.2021.795030)
Supplement: Supplementary Table 1 — The findings from studies exploring consequences of optogenetic interventions on ethanol consumption in two different behavioral paradigms. Blue light stimulations with distinct patterns (tonic and phasic) were conducted through chronic implantable optic fibers, which were connected to a laser of 473 nm with a 100-mW power output. The star (*) indicates that intake measures were performed 3 h following optical stimulations conducted in in high ethanol drinking mice in their home cages. In all other experiments, the stimulations were applied within drinking sessions. [file Table_1.pdf]

Drinking paradigm	Species	Transmitter and pattern	Targeted brain areas	Effect on ethanol intake	Authors
Two bottle choice	Rats	DA / tonic	VTA	Decrease	Bass et al., 2013
Two bottle choice	Mice	DA / tonic	VTA	No effect*	Juarez et al., 2017
Two bottle choice	Rats	DA / phasic	VTA	No effect	Bass et al., 2013
Two bottle choice	Mice	DA / phasic	VTA	Decrease*	Juarez et al., 2017
Operant behavior	Rats	NE / tonic	LC	Increase	Deal et al., 2020
Operant behavior	Rats	NE/ phasic	LC	Decrease	Deal et al., 2020
